# Effects of tibial and parasacral nerve electrostimulation techniques on women with poststroke overactive bladder: study protocol for a randomized controlled trial

**DOI:** 10.1186/s13063-020-04856-4

**Published:** 2020-11-19

**Authors:** Thais Alves Candido, Bruna Miranda Ribeiro, Cristiane Rodrigues Cardoso de Araújo, Rogério de Melo Costa Pinto, Ana Paula Magalhães Resende, Vanessa Santos Pereira-Baldon

**Affiliations:** Federal University of Uberlândia, R. Benjamin Constant, 1286 - Nossa Sra. Aparecida, Uberlândia, MG 38400-678 Brazil

**Keywords:** Stroke, Physical therapy, Electrostimulation, Women’s health, Neurogenic urinary bladder

## Abstract

**Background:**

Neurogenic bladder (NB) can affect people after stroke episodes. NB features changes in the normal voiding pattern at the bladder filling and emptying phases. Overactive NB is characterized by urgency symptoms, with or without urinary incontinence, caused by NB. This disorder affects many domains of life (physical, social, psychological, domestic, sexual) that limit personal autonomy and degrade the quality of life. Among the several treatments available, the conservative physical therapy intervention through tibial nerve electrostimulation (TNES) and parasacral electrostimulation (PSES) can help improve patient conditions with a smaller number of collateral effects than those of interventions based on medication. The aim of the present study is to compare the effects of TNES and PSES techniques in women with overactive NB after stroke episodes to assess the impact of urinary incontinence in these women, on their number of incontinence episodes, daytime and nocturnal urinary frequency, and quality of life.

**Methods:**

This is a prospective clinical study to compare two randomized groups based on parallel and blind conditions. Forty-four women who have had a stroke episode at least 30 days before the trial and who have developed overactive NB will be recruited for the trial. All patients will be subjected to initial evaluation and randomly divided into two groups, TNES and PSES. Subsequently, the two groups will be subjected to a 12-session intervention protocol, twice a week. A new evaluation will be performed after the intervention.

**Discussion:**

The results of this study will contribute to the physiotherapeutic treatment of women with NB after a stroke episode since such results will add information about the benefits of this treatment, urinary control, and the improvements in the quality of life of these women.

**Trial registration:**

Brazilian Registry of Clinical Trials (REBEC) RBR-2bn2z4. Registered on December 11, 2018

## Administrative information

Note: the numbers in curly brackets in this protocol refer to SPIRIT checklist item numbers. The order of the items has been modified to group similar items (see http://www.equator-network.org/reporting-guidelines/spirit-2013-statement-defining-standard-protocol-items-for-clinical-trials/).

**Table:** 

Title {1}	Effects of tibial and parasacral nerve electrostimulation techniques on women with poststroke overactive bladder: study protocol for a randomized controlled trial
Trial registration {2a and 2b}.	Brazilian Registry of Clinical Trials (REBEC), registration RBR-2bn2z4
Protocol version {3}	Version 2 (October 14, 2019)
Funding {4}	Coordenação de Aperfeiçoamento de Pessoal de Nível Superior (CAPES), Brazil – Finance Code 001. There is no direct funding to the project. Capes finances postgraduate programs in Brazil. Thus, the financing of research-related expenses was the responsibility of the team of researchers involved in the project.
Author details {5a}	Federal University of Uberlândia, Brazil
Name and contact information for the trial sponsor {5b}	No sponsor
Role of sponsor {5c}	Not applicable

## Introduction

### Background and rationale {6a}

Stroke episodes result from the fast development of clinical signs of focal and/or global disorders in brain function. These disorders have ischemic or hemorrhagic vascular origin; therefore, they cause changes in the cognitive and sensory-motor planes, depending on the injured area [1]. Vesico-sphincter disorders can affect people after stroke episodes. Neurogenic bladder (NB) is the most common of these disorders since it can change normal urinary patterns at the bladder filling and emptying phases [2].

NB is featured by normal bladder function losses caused by injuries in parts of the central nervous system or in peripheral nerves involved in urinary control, which can account for changes in such functions and result in hyperactive or hypoactive bladder [2]. Overactive NB is characterized by urgency, with or without urgency urinary incontinence, usually with increased daytime frequency and nocturia in the setting of a clinically relevant neurologic disorder with at least partially preserved sensation [3].

Urinary incontinence (UI) after a stroke episode is frequent; in addition, UI is a strong predictor of poorly functioning prognostics [4]. Stroke-related compromised urinary function depends on the degree, size, and site of the injury, but approximately 80% of UI cases can affect the lower urinary tract [2]. UI affects several domains of life (physical, social, psychological, domestic, and sexual) since it limits personal autonomy and degrades the quality of life [4]. Diagnosis and the correct treatment of bladder dysfunction are essential to improve patients’ well-being, to prolong their survival, and to reduce sequels [2].

Using anticholinergic medication to treat hyperactive NB improves disease symptoms [5], but its long-term use is limited by patients’ tolerance to the treatment [6]. Previous studies have reported collateral effects such as intestinal constipation [6, 7], dry mouth, heat intolerance [5], and incomplete urination [6]. Botulinum toxin A is another option to treat NB. It can be used to treat vesico-sphincteric dyssynergia [8]; however, the cost of this treatment is high, and its duration is limited to 6–9 months [5]. Thus, it is necessary to identify new therapeutic options to replace lower-cost treatments applied to overactive NB symptoms—these options must not trigger systemic collateral effects or compromise bladder emptying [6].

Physiotherapeutic treatment through electrostimulation of the tibial and parasacral nerves has been seen as an option to treat overactive bladder in adult [9–12] and infant [13, 14] patients. However, little is known about the results of these techniques between patients who present NB after stroke episodes. Monteiro et al. [15] observed that electrostimulation of the tibial nerve reduced urinary urgency and frequency in men after stroke episodes. On the other hand, Silva et al. [16] did not find statistically significant differences before and after this treatment in four women who presented NB after a stroke episode in a series of cases.

Given the great number of stroke carriers with overactive NB symptoms and the loss in their quality of life, it is necessary to investigate the most effective conservative treatments against such disorder. It is essential to highlight that, although these techniques are often used in clinical practice, there are no studies comparing the electrostimulation of these two nerves in overactive NB treatment after a stroke episode. In addition, few studies that have analyzed these techniques presented low methodological quality. The analysis of the effects of these techniques on women presenting overactive NB after strokes allows for the adoption of a more accurate treatment to rehabilitate these women. This treatment aims at improving the quality of life of such women since it is of easy application and well accepted by patients, as well as fewer reports of side effects.

### Objectives {7}

The aim of the present randomized trial is to compare the effects of tibial nerve electrostimulation (TNES) and parasacral electrostimulation (PSES) techniques in women with overactive NB after stroke to UI impact on the lives of these patients, to urinary frequency, to the number of urinary losses, and to quality of life. We hypothesize that both groups will present fewer symptoms and that there will be no differences between groups.

### Trial design {8}

This is a single-blind randomized controlled trial to compare two randomized groups in parallel (1,1). This article has been written in accordance with the SPIRIT (Standard Protocol Items: Recommendations for Interventional Trials) guidelines.

## Methods: participants, interventions, and outcomes

### Study setting {9}

The trial will be carried out in the facilities of the Sports and Physical Therapy School in the Laboratory of Kinesio-Functional Pelvic Development and Women’s Health of the Federal University of Uberlândia, Brazil.

### Eligibility criteria {10}

Participants presenting the following features will be included in the trial:

- Age group 40–70 years;
- Ischemic or hemorrhagic stroke;
- To have had a stroke episode at least 30 days before the trial;
- Overactive bladder after a stroke episode, proven in the medical report;
- Lack of infection in the urinary tract and bladder tumor; and
- Absence of cognitive deficits [17]. The instrument known as Mini-Mental State Examination (MMSE) will be applied to indicate whether the patient presents some traces of cognitive impairment at the initial evaluation. The instrument is divided into seven dimensions, including time orientation, spatial orientation, immediate memory, attention, calculation, evocation, language, and visual construction. The total MMSE score ranges from 0 to 30 points. The instrument was validated to Portuguese from Brazil [18]. Only participants who reach values above 18 will be included in the study [17].

#### Exclusion criteria

Patients presenting the following features will be excluded from the trial:

- Pregnant women;
- Women who already presented UI before a stroke episode;
- Women who have a pacemaker implant;
- Women who use medication to treat NB; and
- Women who have undergone medical treatment with botulinum toxin to treat NB.

### Who will take informed consent? {26a}

The signed informed consent form will be obtained from each patient prior to their participation in the study. The evaluator will acquire consent during the inclusion visit. The evaluator will explain all stages of the study, and the volunteers who agree to participate will sign the term.

### Additional consent provisions for the collection and use of participant data and biological specimens {26b}

Not applicable. There will be no biological sample collection in this study.

## Interventions

### Explanation for the choice of comparators {6b}

The techniques of TNES and PSES are noninvasive and widely used in clinical practice. No studies were found using this technique in women with NB after stroke. Therefore, we would such as to know which one is more effective for the treatment of poststroke women.

### Intervention description {11a}

The treatment of both groups will be performed twice a week for 6 weeks, totaling 12 sessions. The choice for the duration of treatment is based on previous studies that have shown the beneficial effects of neuromodulation caused by TNES after a mean treatment time of 12 sessions, twice a week. The present randomized controlled trial will be based on two sessions per week, given the belief that such treatment time would intensify the effects of electrostimulation on neuronal plasticity [11, 12].

**Fig. 1:**
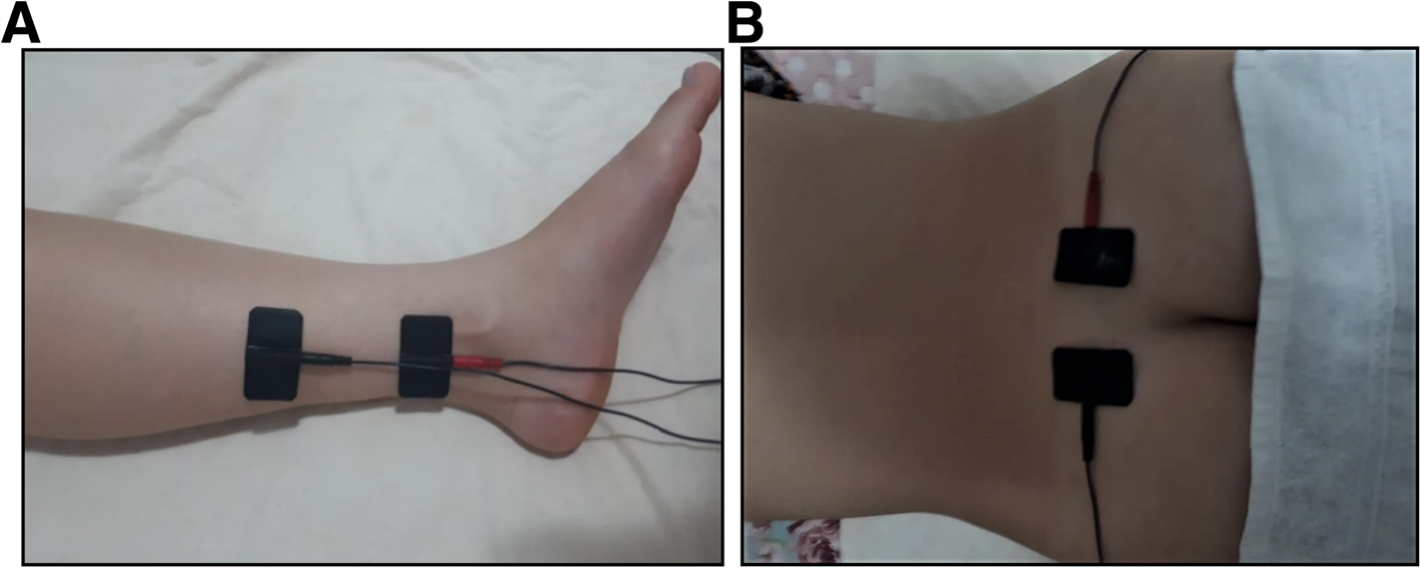
Images of electrode positions on the posterior tibial (**a**) and parasacral nerves (**b**)

Participants in the TNES group will be placed in the lateral decubitus position and the electrodes will be placed on the tibial region of the limb—counterlateral to the hemiplegic/hemiparetic side. One electrode will be placed posterior to the medial malleolus and another one will be placed approximately 10 cm above it (belly region of the posterior tibialis muscle). The participants in the PSES group will be positioned to achieve the greatest comfort. Two superficial electrodes will be placed on each side of S3 and S2 to directly stimulate spinal reflexes and reach suprasacral centers, as described in other studies [7] (Fig. 1).

**Fig. 2:**
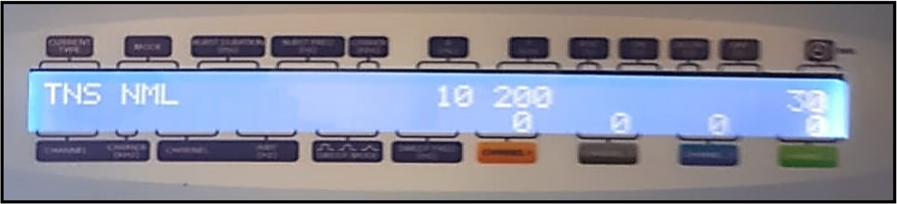
Protocol used in the Neurodym Multibrand device, IBRAMED®, in both groups

Transcutaneous electrical nerve stimulation (TENS) was performed in the device model Neurodym Multibrand, IBRAMED®. Parameters applied to the TNES and PSES groups will be the same: wave frequency (F) at 10 Hz variation and pulse length (T) of 200 μs for 30 min [5, 12] (Fig. 2). This frequency was chosen because Jiang and Lindström [19] have shown that 10 Hz is the best frequency to cause bladder inhibition in mice. The current intensity in both groups will be increased according to the patients’ sensitivity limit.

### Criteria for discontinuing or modifying allocated interventions {11b}

The criterion for discontinuation is the withdrawal of patient consent prior to publication of the results.

### Strategies to improve adherence to interventions {11c}

To improve adherence, telephone contact will be made with the volunteers the day before the intervention session to remind them of their appointment.

### Relevant concomitant care permitted or prohibited during the trial {11d}

During the protocol, medical consultations and the intake of medications are allowed, with the exception of medication for the treatment of NB.

### Provisions for posttrial care {30}

Researchers are responsible for any harm that occurs as a result of the study. Thus, the researchers will provide for participants’ health care needs that arise as a direct consequence of trial participation.

### Outcomes {12}

#### Primary outcome measure

The primary outcome measure is any changes in the International Consultation on Incontinence Questionnaire-Short Form (ICIQ-SF) from baseline to final evaluation. The ICIQ-SF questionnaire is simple and fast (only four questions), and it will be used for UI impact evaluation. This questionnaire has general scoring ranging from 0 to 21 points; the closer to 21 points the score is, the stronger the impact of urinary losses on quality of life [11, 20].

#### Secondary outcome measures

The secondary outcomes of the study are urinary frequency during the day and night, number of incontinence episodes, and quality of life. A bladder diary was used to assess urinary habits in order to quantify daytime and nocturnal urination frequency and episodes of incontinence. Volunteers will be oriented to fulfill a table with this information, provided by the researchers, for 3 days [21].

The World Health Organization Quality of Life – BREF (WHOQOL-BREF) questionnaire will be used to assess the quality of life before and after the intervention. This instrument was developed by the World Health Organization and is based on the quality of life concept, which encompasses positive (medication mobility, played role, satisfaction) and negative points (fatigue, pain, medication dependence, and negative feelings) [22]. The questionnaire comprises 26 questions with 5 coordinated options. There are 24 questions in the four domains (physical, psychological, social relationships, and environment) composing the original instrument and two general questions regarding the quality of life [23, 24]. Each specific domain is assessed based on values found through the WHOQOL-BREF—the higher the score is (maximum = 100), the better the quality of life related to that domain [25].

### Participant timeline {13}

**Fig. 3:**
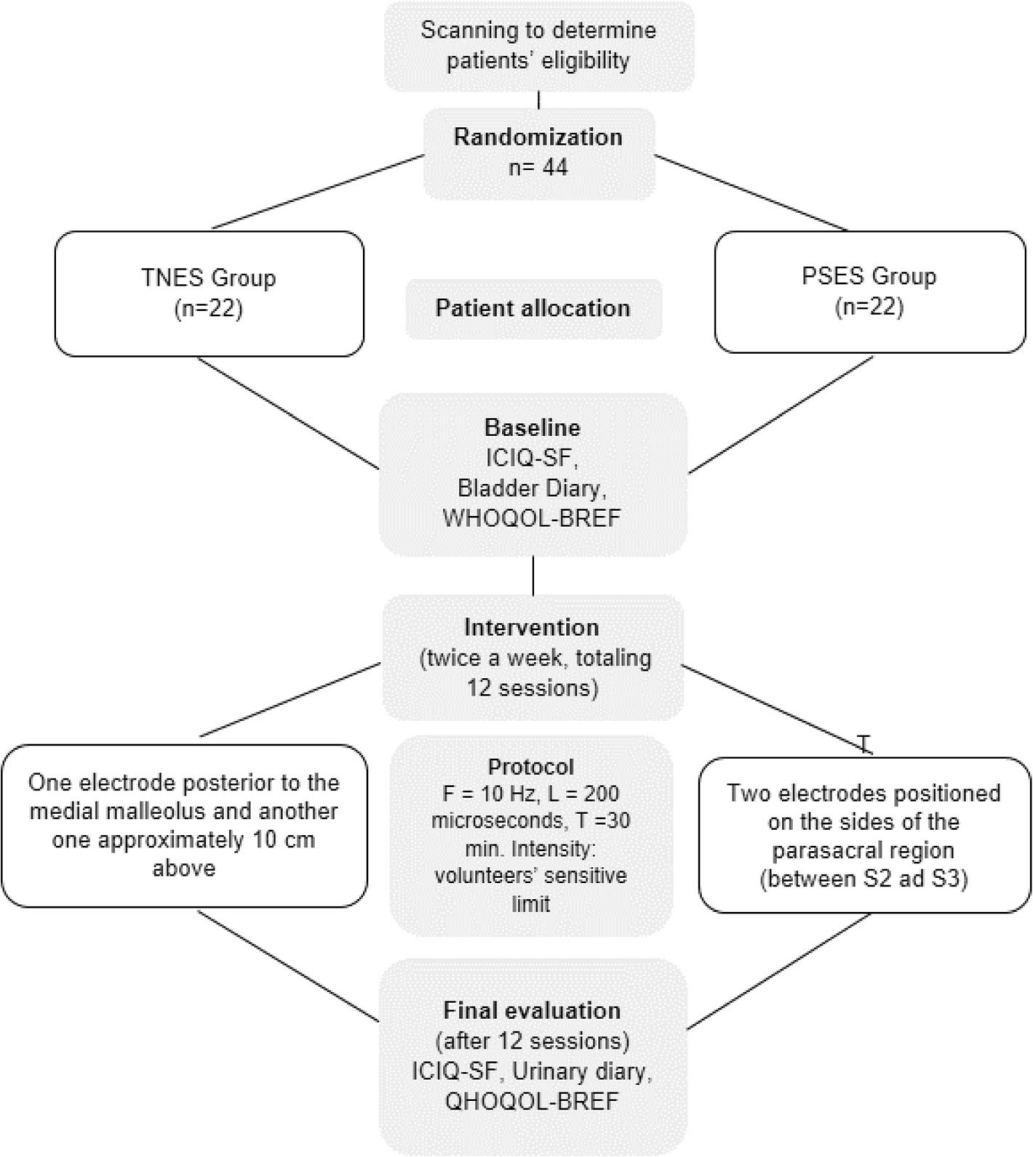
Flow diagram of the trial design

All assessments will be carried out at baseline and after 12 intervention sessions. The schematic diagram of the participant timeline can be seen in Fig. 3.

### Sample size {14}

Studies that applied the techniques to other populations were analyzed to estimate the percentage of improvement in urinary symptoms after application of TNES and PSES. The studies of Malm-Buatsi et al. [26], Monteiro et al. [15], and Kabay et al. [27] were analyzed. Thus, considering a confidence of 99%, a power of the test of 90%, considering the proportion of patients who had improvement after TNES of 69% (PA = 0.69) and patients who had improvement after PSES of 13% (PB = 0.13), the sample size for each group was 22 women. To calculate the sample, Bioestat 5.0 software was used [28].

### Recruitment {15}

Volunteers from the community in Uberlândia, Brazil, will be recruited for the trial after information about the research is publicized. The recruitment information will be carried in newspaper and radio announcements, e-mails, social networks, and visitations to the rehabilitation center in the city.

## Assignment of interventions: allocation

### Sequence generation {16a}

Participants will be randomized in a 1:1 ratio to the TPES or PSES group by computer-generated random numbers.

### Concealment mechanism {16b}

A researcher not involved in the data collection will assign the groups by the sealed envelope method. The opaque envelopes will be numbered and kept locked away, available only to the study researchers.

### Implementation {16c}

After including the participant, the researcher responsible for the interventions will open the envelope with the lowest number available to determine which group the participant belongs to.

## Assignment of interventions: blinding

### Who will be blinded? {17a}

The researcher in charge of the interventions to be applied to the patients will be blind to the assessments. The researcher in charge of the initial and final evaluations and the one accounting for data processing will be blind for treatment allocation. Patients will not be blind to the procedure because of the difficulties set by differences between techniques.

### Procedure for unblinding if needed {17b}

The loss of blinding should not be carried out unless there is a threat to the participant’s safety as a consequence of the study procedures.

## Data collection and management

### Plans for assessment and collection of outcomes {18a}

A trained researcher will perform all assessments at baseline and after 12 sessions. The Portuguese version of the ICIQ questionnaire presents satisfactory reliability and construct validity [20], which is a questionnaire widely used in research and clinical practice because it is robust, brief, and simple [29].

The 3-day bladder diary was chosen because its reliability is efficient regarding the number of micturition episodes per day and episodes of urgency in patients suffering from neurogenic urinary dysfunction [30].

Fleck et al. [23] demonstrated that the Portuguese version of the WHOQOL-BREF showed a good performance concerning internal consistency, discriminant validity, criterion validity, concurrent validity, and test-retest reliability. Therefore, this questionnaire allies good psychometric performance and practicality for use.

### Plans to promote participant retention and complete follow-up {18b}

Intention-to-treat analysis will be carried out [31]. Therefore, all participants who discontinue or deviate from the initially randomized intervention will be evaluated. Those participants who discontinue the intervention will receive telephone contact from the researcher after 6 weeks of the first evaluation to attend the laboratory for reevaluation.

### Data management {19}

Following the institution’s protocols, all data will be considered anonymous, and participants will be identified by numbers. All source documents will be stored in locked file cabinets with secure and limited access. The data will be transferred to a secure online data cloud through double-checking between researchers. Only the research group has an individual password to access the data.

### Confidentiality {27}

The study will be conducted in accordance with Brazilian rules and regulations. All data generated in this study will remain confidential. Only the research team has access to the study data. Access to data will only be provided in the event of audits or regulatory regulation by the institution.

### Plans for collection, laboratory evaluation, and storage of biological specimens for genetic or molecular analysis in this trial/future use {33}

Not applicable as no biological samples will be collected.

## Statistical methods

### Statistical methods for primary and secondary outcomes {20a}

Statistical Package for the Social Sciences (SPSS Statistics version 23) software will be used in the statistical analysis. The Shapiro-Wilk test will be initially applied to assess data normality. Data will be described as the mean (standardized deviation) for normally distributed continuous variables, median (interquartile range) for continuous variables without normal distribution, and frequency (percentage) for categorical variables.

For parametric data, comparison between the groups will be carried out using the analysis of variance (ANOVA) test. Significance values lower than 0.05, at the 95% confidence interval, will be interpreted as statistically significant. The clinical relevance of recorded values will be confirmed through effect-size calculations (Cohen’s *d*) based on significant differences. The following effects will be taken into account: 0.00 to 0.49, low; 0.50 to 0.79, medium; and above 0.80, high [32]. The intention-to-treat analysis will be carried out [31].

### Interim analyses {21b}

No interim analysis will be performed.

### Methods for additional analyses (e.g., subgroup analyses) {20b}

None planned.

### Methods in analysis to handle protocol non-adherence and any statistical methods to handle missing data {20c}

The analysis will be performed according to the intention-to-treat criteria. Any missing data and the reason for the missing data will be described for each group.

### Plans to give access to the full protocol, participant-level data, and statistical code {31c}

The datasets analyzed during the study will be available from the corresponding author upon reasonable request.

## Oversight and monitoring

### Composition of the coordinating center and trial steering committee {5d}

The researchers involved in the study (see on the title page) form the coordinating center responsible for study coordination, monitoring, data acquisition and management, and statistical analysis.

### Composition of the data monitoring committee, its role, and reporting structure {21a}

This study will not have a data monitoring committee, as it is a short-term trial with minimal known risks.

### Adverse event reporting and harm {22}

This study involves minimal known risks but, daily, the researcher responsible for the interventions will question the participants about possible adverse effects. It is possible to complain of skin irritation due to the electrode fixation or even increased symptoms. Any harm detected will be reported to the institution’s research ethics committee.

### Frequency and plans for auditing trial conduct {23}

No audits are planned because this trial is academic.

### Plans for communicating important protocol amendments to relevant parties (e.g., trial participants, ethical committees) {25}

According to national regulations, major modifications of the protocol require a formal amendment to the protocol and are to be approved by the institution’s research ethics committee and modified in the Brazilian Registry of Clinical Trials.

### Dissemination plans {31a}

The trial results will be submitted for publication in relevant journals and presented at conferences in the area of physiotherapy and urology. In addition, the results will be published on the university’s social media using accessible language so that it is known to the population.

## Discussion

Electrostimulation techniques are widely used to treat UI [33] and, in Brazil, it is often used by physical therapists in their clinical practice. However, studies about the herein assessed specific population—women with overactive NB after a stroke episode subjected to the parasacral technique—were not found. Only one study involving a sample of 4 women subjected to the tibial nerve technique was found in the literature [16]. There are no clinical essays based on the protocol and that have compared the effects of these two techniques in this population. Accordingly, the results of this first study with the application of these techniques in the population of women after a stroke are able to assist the clinical decision-making of physical therapists. As a technique with low cost, no side effects, and good adherence of patients [6], electrostimulation can assist in reducing urinary complaints of patients. Moreover, these results can contribute to new studies based on comparing treatment techniques to improve the quality of life of these patients.

### Trial status

The study was registered at the Brazilian Registry of Clinical Trials (REBEC) (number RBR-2bn2z4) on December 11, 2018. Recruitment began on January 09, 2019. For organizational issues of intervention services in women after strokes, it was necessary to conduct the recruitment of volunteers before sending the protocol. The study was interrupted due to the COVID-19 pandemic, and recruitment is expected to be completed by 2021.

## Abbreviations

ANOVA: Analysis of variance
F: Frequency
ICIQ: International Consultation on Incontinence Questionnaire-Short Form
MMSE: Mini-Mental State Examination
NB: Neurogenic bladder
PSES: Parasacral electrostimulation
REBEC: Brazilian Registry of Clinical Trials
SPIRIT: Standard Protocol Items: Recommendations for Interventional Trials
T: Pulse length
TNES: Tibial nerve electrostimulation
UI: Urinary incontinence
WHOQOL-BREF: World Health Organization Quality of Life – BREF
